# A 13-Gene Metabolic Prognostic Signature Is Associated With Clinical and Immune Features in Stomach Adenocarcinoma

**DOI:** 10.3389/fonc.2021.612952

**Published:** 2021-06-21

**Authors:** Zaisheng Ye, Miao Zheng, Yi Zeng, Shenghong Wei, He Huang, Yi Wang, Qinying Liu, Zhitao Lin, Shu Chen, Qiuhong Zheng, Luchuan Chen

**Affiliations:** ^1^ Department of Gastrointestinal Surgical Oncology, Fujian Cancer Hospital & Fujian Medical University Cancer Hospital, Fuzhou, China; ^2^ Department of Clinical Laboratory, Fujian Provincial Maternity and Children Hospital, Affiliated Hospital of Fujian Medical University, Fuzhou, China; ^3^ Department of Clinical Laboratory, Fujian Provincial Maternity and Child Health Hospital, Affiliated Hospital of Fujian Medical University, Fuzhou, China; ^4^ Department of Digestive Endoscopy, Fujian Cancer Hospital & Fujian Medical University Cancer Hospital, Fuzhou, China

**Keywords:** stomach adenocarcinoma, metabolism-based prognostic signature, clinical characteristics, tumor microenvironment, biomarker

## Abstract

Patients with advanced stomach adenocarcinoma (STAD) commonly show high mortality and poor prognosis. Increasing evidence has suggested that basic metabolic changes may promote the growth and aggressiveness of STAD; therefore, identification of metabolic prognostic signatures in STAD would be meaningful. An integrative analysis was performed with 407 samples from The Cancer Genome Atlas (TCGA) and 433 samples from Gene Expression Omnibus (GEO) to develop a metabolic prognostic signature associated with clinical and immune features in STAD using Cox regression analysis and least absolute shrinkage and selection operator (LASSO). The different proportions of immune cells and differentially expressed immune-related genes (DEIRGs) between high- and low-risk score groups based on the metabolic prognostic signature were evaluated to describe the association of cancer metabolism and immune response in STAD. A total of 883 metabolism-related genes in both TCGA and GEO databases were analyzed to obtain 184 differentially expressed metabolism-related genes (DEMRGs) between tumor and normal tissues. A 13-gene metabolic signature (*GSTA2*, *POLD3*, *GLA*, *GGT5*, *DCK*, *CKMT2*, *ASAH1*, *OPLAH*, *ME1*, *ACYP1*, *NNMT*, *POLR1A*, and *RDH12)* was constructed for prognostic prediction of STAD. Sixteen survival-related DEMRGs were significantly related to the overall survival of STAD and the immune landscape in the tumor microenvironment. Univariate and multiple Cox regression analyses and the nomogram proved that a metabolism-based prognostic risk score (MPRS) could be an independent risk factor. More importantly, the results were mutually verified using TCGA and GEO data. This study provided a metabolism-related gene signature for prognostic prediction of STAD and explored the association between metabolism and the immune microenvironment for future research, thereby furthering the understanding of the crosstalk between different molecular mechanisms in human STAD. Some prognosis-related metabolic pathways have been revealed, and the survival of STAD patients could be predicted by a risk model based on these pathways, which could serve as prognostic markers in clinical practice.

## Introduction

Stomach adenocarcinoma (STAD) accounts for 95% of stomach tumors, which is associated with high mortality (1). The most effective treatment is radical surgery in the early stages combined with chemotherapy, postoperative radiotherapy, and lymphadenectomy, but 65% of patients with STAD presented at an advanced stage, and nearly 85% of patients with STAD display lymph node metastasis at the time of diagnosis (2). Despite the decreasing incidence worldwide, the 5-year survival rate of patients with resectable STAD ranges from 10% to 30% (3). Although STAD can be treated with radical surgery and adjuvant therapy, more than 40% of patients continue to experience recurrence or tumor metastasis (4). The association between microarray-based gene expression profiling and the corresponding phenotypic changes in STAD has allowed accurate early diagnosis or evaluation of prognosis (5). The development of novel biomarkers in STAD would aid early diagnosis, guide surgical and adjuvant therapy decision making, and provide potential therapeutic targets.

Changes in metabolism-related genes result in abnormal metabolism-related pathways and the production of metabolites in cancer cells, which are associated with transformation, tumor growth, and tumor progression (6). Specific metabolic activities have been developed to image tumors, provide prognostic biomarkers, and identify therapeutic targets (7). Thus, exploring and exploiting specific metabolic alterations in cancer has implications for clinical oncology and basic cancer pathophysiology. For example, increasing evidence has shown that the disordered metabolism of non-essential amino acids plays a key role in cancer development and progression *via* metabolic reprogramming in cancer cells (8). Proline metabolism in cancer, which is involved in collagen synthesis and degradation, influences tumor heterogeneity and the epigenetic landscape (9). Extensive crosstalk has being revealed between abnormal glucose metabolism and cancer cell signaling, a great example of which is the “Warburg effect” (aerobic glycolysis) (10). Additionally, the metabolism of ketone bodies, fatty acids, and choline is also significantly altered in cancer cells (11). These exciting advancements in cancer metabolism reprogramming and crosstalk have facilitated the identification of new targets for treating malignancies (12). The development of immunotherapy has resulted in a fundamental change in the survival rate and prognosis of cancer (13). Furthermore, the association between the immune microenvironment and other biological processes has become increasingly important for immunotherapy. The immunoediting theory suggests that various metabolic machinery influence the behavior of immune cells and antitumor immune responses (14). Metabolic stress in tumor-infiltrating immune cells leads to changes in their functional activities, thereby promoting the evasion of immunosurveillance by cancer cells (15). Thus, studies on metabolic reprogramming of the immune microenvironment would promote the repurposing of drugs targeting cancer metabolism and immunotherapy.

Recently, with the rapid development of bioinformatics, many novel biomarkers have been discovered for the diagnosis and prognosis of multiple cancers based on large-scale RNA-sequencing (RNA-seq) transcriptome data and the corresponding clinical follow-up information. More efficient and accurate approaches have promoted the application of personalized medicine in clinical practice. With the discovery of complex biological processes in cancer, using a gene set to construct a prognostic signature would be better than a single gene pattern. In this study, a metabolism-based prognostic signature was systematically analyzed by combining data from the TCGA and GEO databases. Patients with STAD were divided into high- and low-risk score groups according to the metabolism-based prognostic risk score (MPRS). This metabolism-based prognostic signature was verified to be significantly associated with survival in STAD using TCGA and GEO data. Furthermore, the significant differences in the distribution of immune cells between the high- and low-risk score groups according to this metabolism-based prognostic signature further revealed the differentially expressed immune-related genes (DEIRGs) between the two groups. This indicates that metabolic reprogramming of the immune microenvironment requires further experimental verification and clinical research.

## Materials and Methods

### Metabolism-Related Genes in STAD

The mRNA expression and corresponding clinical data were downloaded from The Cancer Genome Atlas (TCGA) website (https://portal.gdc.cancer.gov/) (16) and the GEO database (https://www.ncbi.nlm.nih.gov/geo/query/acc.cgi?acc=GSE84437). The type of gene expression data was FPKM. A total of 407 and 433 samples were obtained from TCGA and GEO (GSE84437), respectively, which included 883 metabolism-related genes in both TCGA and GEO databases based on the Molecular Signatures Database (https://www.gsea-msigdb.org/gsea/msigdb/index.jsp). The clinical characteristics based on the TCGA database included sex (male and female), age (35 to 90 year), grade (including grades 1, 2, and 3), pathologic T (tumor size, including T1, T2, T3, T4, and TX), pathologic M (tumor metastasis, including M0, M1, and MX), pathologic N (tumor lymph node metastasis, including N0, N1, N2, and NX), pathologic stage (stages I, II, III, and IV), and survival data (survival time and survival status). The clinical characteristics based on the GEO database included sex (male and female), age (27 to 86 years old), pathologic T (tumor size, including T1, T2, T3, and T4), and pathologic N (tumor lymph node metastasis, including N0, N1, N2, and N3). There are two human tasks for auditor 1 and auditor 2, respectively, to take actions and reviewed the data of included samples to determine the next actions according to the auditors’ input.

### Identification of DEMRGs Between Normal and Tumor Tissues in STAD

The ‘limma’ package (https://www.bioconductor.org/packages/release/bioc/html/limma.html) was used to identify DEMRGs between normal and tumor tissues in STAD (p < 0.05, false discovery rate (FDR) ≤ 0.05, fold change ≥ 2) from the TCGA database. The p value was adjusted by FDR.

### Functional and Pathway Enrichment Analyses of DEMRGs in STAD

The identified DEMRGs in STAD from TCGA data were enriched in various Kyoto Encyclopedia of Genes and Genomes (KEGG) pathways (p < 0.05 and FDR < 0.05) according to the DAVID functional annotation bioinformatics microarray database (https://david.ncifcrf.gov/) (17). Analysis of gene ontology (GO) terms was performed using Cytoscape ClueGO (adjusted P < 0.05, corrected using the Benjamini-Hochberg method) based on the subtype analysis of biological processes (BPs) (18). All DEMRGs were identified using the protein-protein interaction (PPI) network based on the STRING database (https://string-db.org/). The criteria of hub molecule searching were set as a molecular complex detection (MCODE) score > 7, and statistical significance was set at P < 0.05 (19).

### Cox Regression and Overall Survival Analyses of DEMRGs in STAD

The Cox proportional hazard regression model was performed using the ‘survival’ package in R (https://www.rdocumentation.org/packages/survival/versions/3.2-3) to select OS-related DEMRGs (P < 0.05) in STAD based on the survival information of TCGA data. Each OS-related DEMRG from Cox regression analysis was further plotted in the Kaplan–Meier survival curve using Kaplan-Meier Plotter (https://kmplot.com/analysis/) (20).

### Lasso Regression Construction and Verification in STAD

The OS-related DEMRGs were used to construct a prognostic model in STAD with lasso regression using the ‘glmnet’ package in R (https://cran.r-project.org/web/packages/glmnet/index.html). Patients with STAD from the TCGA group were divided into high- and low-MPRS groups according to the median value of MPRS (median value = −0.39). Similarly, patients with STAD from the GEO group were divided into high and low MPRS groups according to the median value of MPRS (median value = −0.39). The receiver operating characteristic (ROC) curves were plotted using the R package (https://www.rdocumentation.org/packages/pROC/versions/1.16.2/topics/roc) to show the specificity and sensitivity of MPRS in the TCGA group. The Kaplan-Meier curve was used to evaluate the relevance of overall survival between the high and low MPRS subtypes. Additionally, univariate and multivariate Cox regression models were used to analyze the association between OS and MPRS in STAD based on some parameters, including age, sex, grade, pathologic stage, pathologic T, pathologic M, pathologic N, and risk score in the TCGA group and age, sex, pathologic T, pathologic N, and risk score in the GEO group. The clinical characteristics and MPRS-based assessment nomogram (https://cran.r-project.org/web/packages/rms/index.html) were used to evaluate prognosis in patients with STAD patients (1-, 2-, and 3-year survival rates) in both the TCGA and GEO groups.

Moreover, gene set enrichment analysis (GSEA) (version 4.1.0) identified different gene sets in the high- and low-MPRS groups by 1,000 permutations p < 0.05, and FDR q-value < 0.05, calculated using the Benjamini-Hochberg multiple testing). Protein expression levels were verified in the Human Protein Atlas (HPA) (https://www.proteinatlas.org/) (21). In order to compare our findings with the previous studies, we searched genes using GenCLiP 3 (http://ci.smu.edu.cn/genclip3/input_enrichment.php#) (22).

### Identification of Different Immune Cells and DEIRGs Between High- and Low-MPRS Subtypes in STAD

The distribution of immune cells (p < 0.05) was investigated between high- and low-MPRS subtypes in STAD tissue samples using the Kolmogorov-Smirnov tests in R (https://stat.ethz.ch/R-manual/R-devel/library/stats/html/ks.test.html) in the TCGA group. Furthermore, the correlation of different immune cells was determined using the ‘Corrplot’ package in R (https://cran.r-project.org/web/packages/corrplot/vignettes/corrplot-intro.html). The ‘limma’ package (https://www.bioconductor.org/packages/release/bioc/html/limma.html) was used to identify DEIRGs (p < 0.05, false discovery rate (FDR) ≤ 0.05, and fold change ≥1.20) between high- and low-MPRS subtypes in the TCGA group. The identified DEIRGs in STAD were input into the DAVID functional annotation bioinformatics microarray database (https://david.ncifcrf.gov/) to analyze significant KEGG pathways (p < 0.05, FDR < 0.05).

### Cell Lines and Cell Culture

STAD cells MKN-45 and AGS, and normal cells GES-1 were purchased from Keibai Academy of Science (Nanjing, China). RPMI-1640 medium (Corning, NY, USA) plus 10% fetal bovine serum (FBS, Gibco) were used to culture those cells with 5% CO2 atmosphere at 37°C.

### RNA Extraction and qRT-PCR

The STAD cells and normal cells (4 × 10^6^) were used to extract total RNA through the following steps: (i) the cells were washed with PBS (3×); (ii) a volume (1 ml) of TRizol Reagent (Invitrogen) was used to lyse cells (10 min, ice); (iii) 200 μl chloroform was added to each tube with sufficient mixing; (iv) after resting for 5 min on ice, they were centrifuged (12,000 r/min, 15 min); (v) the same volume of isopropanol was added to supernatant with sufficient mixing; (vi) after resting for 15 min on ice, they were centrifuged (12,000 r/min, 15 min); (vii) a volume (1 ml) of ethanol (v/v = 75%) was added to precipitate, and then centrifuged (12,000 r/min, 5 min); and (viii) after removing ethanol, 20μl RNA enzyme-free water was added to dissolve RNA precipitate. Each total RNA was reversely transcribed into cDNA for quantitative real-time PCR (qRT-PCR) analysis with SYBR Premix ExTaq kit (TaKaRa). For the reverse transcription reaction system: (i) add 2 μl 5× gDNA Eraser buffer, 1 μl 5× gDNA Eraser buffer, 500 ng total RNAs, and RNase-free water up to 10 μl at 42°C for 2 min. (ii) Add 1 μl PrimeScript RT Enzyme Mix I, 1 μl RT Primer Mix, 2 μl 5× Prime Script buffer, 4 μl RNase-free water to reaction solution from the first step at 37°C for 15 min, 85°C for 5 s, and save at 4°C. qRT-PCR reaction system contained 5 μl SYBR buffer, 4 μM primers (forward and reverse primers), 2 μl RNase-free water, and 1 μl cDNA. Beta-actin was set as an internal control for gene quantification. The numbers of technical and biological replicates were at least three times for each gene with qRT-PCR analysis.

## Results

### Discovery of DEMRGs Between Tumor and Normal Tissues in STAD Based on TCGA Data

**Figure 1:**
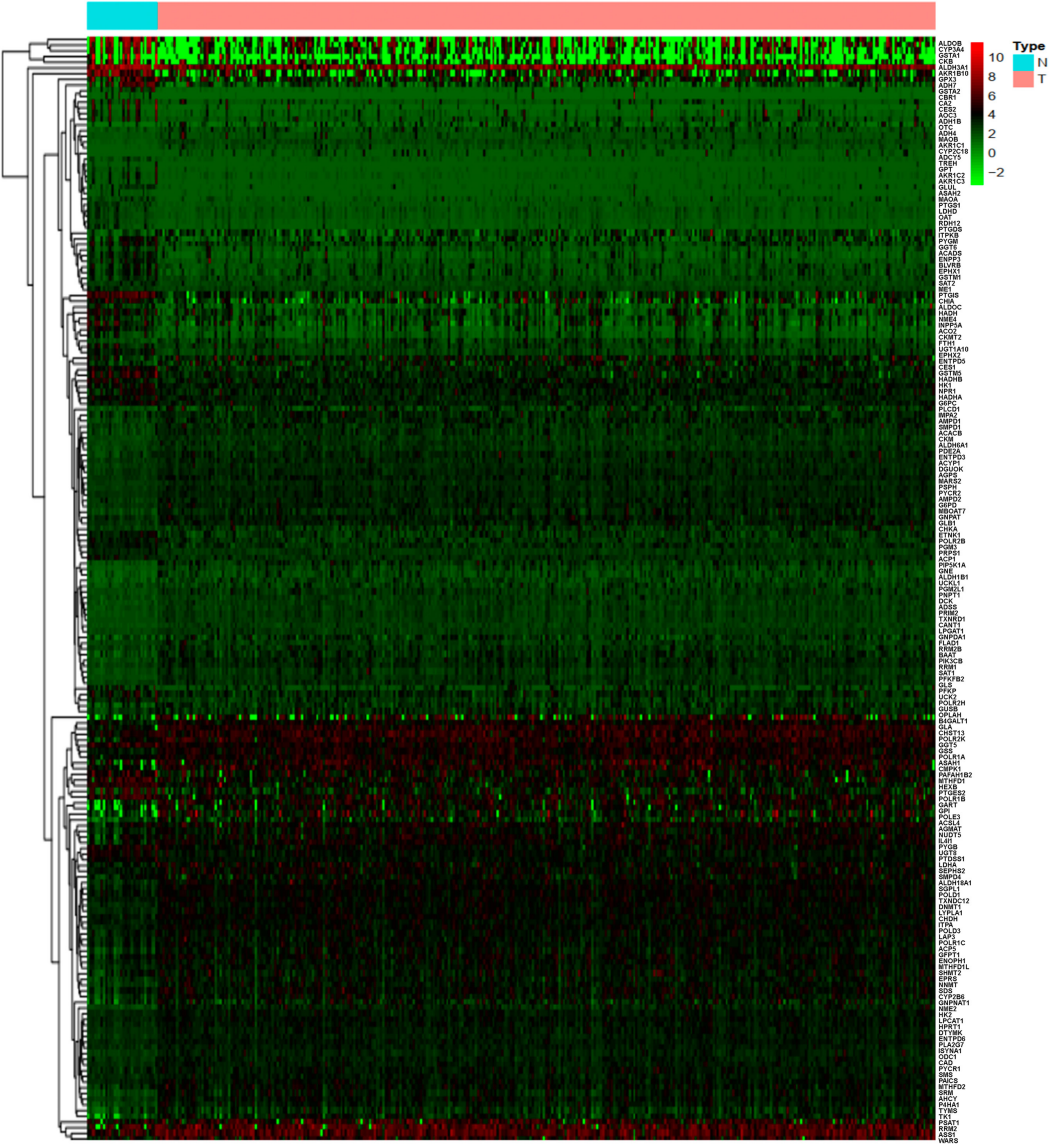
Heatmap of the differentially expressed metabolism-related genes (DEMRGs) between normal and tumor issues in stomach adenocarcinoma [(STAD) (N, normal tissues; T, tumor issues].

A total of 884 MRGs overlapped between the TCGA (**Supplementary Table 1**) and GEO groups (**Supplementary Table 2**). Analysis of DEMRGs between tumor and normal tissues in STAD was performed in the TCGA group. Finally, 184 DEMRGs were identified as DEMRGs between tumor and normal tissues based on TCGA data (**Figure 1** and **Supplementary Table 3**). Among them, 70 DEMRGs were downregulated, and 114 DEMRGs were upregulated (**Figure 1**).

### DEMRGs Were Significantly Enriched in Cancer-Related Pathways and Biological Processes in STAD

**Figure 2:**
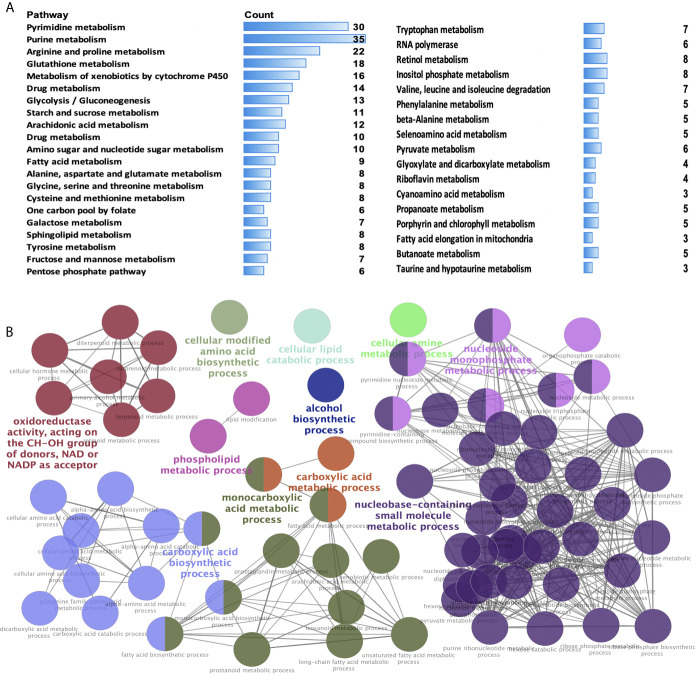
Significant Kyoto Encyclopedia of Genes and Genomes (KEGG) pathways and biological processes (BPs) of differentially expressed metabolism-related genes (DEMRGs) in stomach adenocarcinoma (STAD). **(A)** Pathways in cancer significantly enriched with DEMRGs in SATD (p < 0.05). **(B)** The DEMRGs were classified according to the BPs (p < 0.05). The DEMRGs with significantly enriched in the pathways are shown with a greater node size. Same color indicates the same functional group. A representative group with the most significant term and lag is highlighted.

KEGG enrichment analysis was used to analyze the pathways involved in the identified DEMRGs. A total of 38 statistically significant KEGG pathways were enriched in STAD (**Figure 2A** and **Supplementary Table 4**), and most pathways were closely associated with metabolism-related pathways, including pyrimidine metabolism, purine metabolism, arginine and proline metabolism, glutathione metabolism, metabolism of xenobiotics by cytochrome P450, drug metabolism, glycolysis/gluconeogenesis, starch and sucrose metabolism, arachidonic acid metabolism, drug metabolism, amino sugar and nucleotide sugar metabolism, fatty acid metabolism, alanine, aspartate, and glutamate metabolism, glycine, serine, and threonine metabolism, cysteine and methionine metabolism, one carbon pool by folate, galactose metabolism, sphingolipid metabolism, tyrosine metabolism, fructose and mannose metabolism, pentose phosphate pathway, tryptophan metabolism, RNA polymerase, retinol metabolism, inositol phosphate metabolism, valine, leucine, and isoleucine degradation, phenylalanine metabolism, beta-alanine metabolism, selenoamino acid metabolism, pyruvate metabolism, glyoxylate and dicarboxylate metabolism, riboflavin metabolism, cyanoamino acid metabolism, propanoate metabolism, porphyrin and chlorophyll metabolism, fatty acid elongation in mitochondria, butanoate metabolism, taurine and hypotaurine metabolism. The hub molecules of these signaling pathways should be considered.

GO enrichment analysis according to BPs was performed to analyze the identified DEMRGs. A total of 86 statistically significant BPs were obtained in STAD (**Figure 2B** and **Supplementary Table 5**), which mainly included the following 11 clusters: carboxylic acid metabolic process, carboxylic acid biosynthetic process, monocarboxylic acid metabolic process, alcohol biosynthetic process, cellular modified amino acid biosynthetic process, cellular lipid catabolic process, oxidoreductase activity (acting on the CH-OH group of donors, NAD or NADP as acceptor), cellular amine metabolic process, nucleobase-containing small-molecule biosynthetic process, nucleoside monophosphate metabolic process, and phospholipid metabolic process. These BP enrichments of the identified DEMRGs have broad implications in STAD cells, influencing cell metabolism.

**Figure 3:**
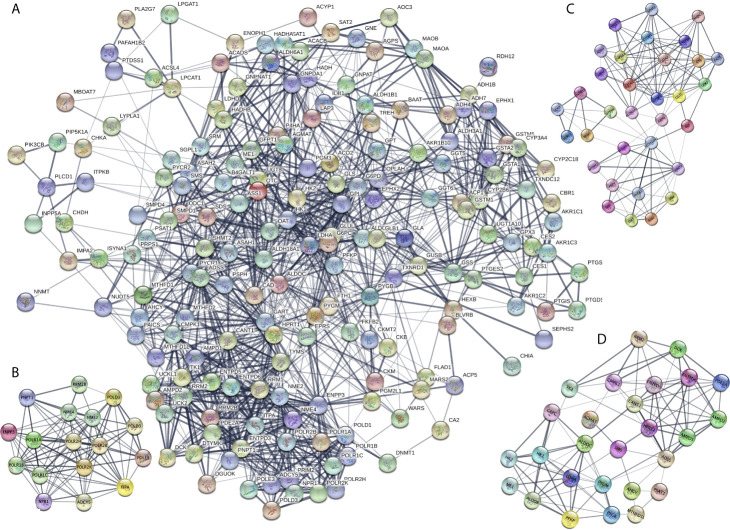
Protein-protein interaction (PPI) network of the differentially expressed metabolism-related genes (DEMRGs) in stomach adenocarcinoma (SATD). **(A)** PPI network of the DEMRGs in SATD. **(B–D)** The entire PPI network was analyzed using MCODE, and three modules (module 1 score = 12.375, module 2 score = 8, and module 3 score = 7. 615) were obtained.

The DEMRGs were identified using the PPI network in STAD (**Figure 3A** and **Supplementary Table 6**). Furthermore, three key modules (module 1 score = 12.375, module 2 score = 8, and module 3 score = 7. 615) were selected (**Figures 3B–D**). Thus, a total of 17 hub molecules were identified in module 1, including POLR1C, PNPT1, POLR2K, POLR1B, POLR1A, POLR2H, RRM2B, POLD1, POLR2B, POLD3, ADCY5, NME4, POLE3, NME2, ITPA, NPR1, and ENPP3 (**Figure 3B**). A total of 33 hub molecules were identified in module 2, including GSTM1, GGT6, GGT5, CYP2B6, GSTA1, ADH4, GSTA2, GPX3, CYP2C18, ADH7, ASS1, SHMT2, GSS, RRM1, MTHFD2, MTHFD1, ALDH3A1, ENTPD3, SRM, SDS, G6PD, CYP3A4, GSTM5, PSPH, ALDH18A1, OAT, DTYMK, UCK2, UCKL1, and GLS (**Figure 3C**). A total of 27 hub molecules were identified in module 3, including DCK, AMPD2, PFKP, LDHA, AMPD1, PSAT1, AGMAT, TK1, RRM2, ME1, HPRT1, SMS, ADSS, CMPK1, HK1, HK2, PYGM, MTHFD1L, ENTPD6, ENTPD5, G6PC, PYGB, CANT1, AHCY, PDE2A, and ALDOB. These identified hub molecules of DEMRGs promoted the understanding of the key molecular mechanisms on metabolism underlying STAD development.

### Survival Analysis of DEMRGs in STAD Based on TCGA Data

**Figure 4:**
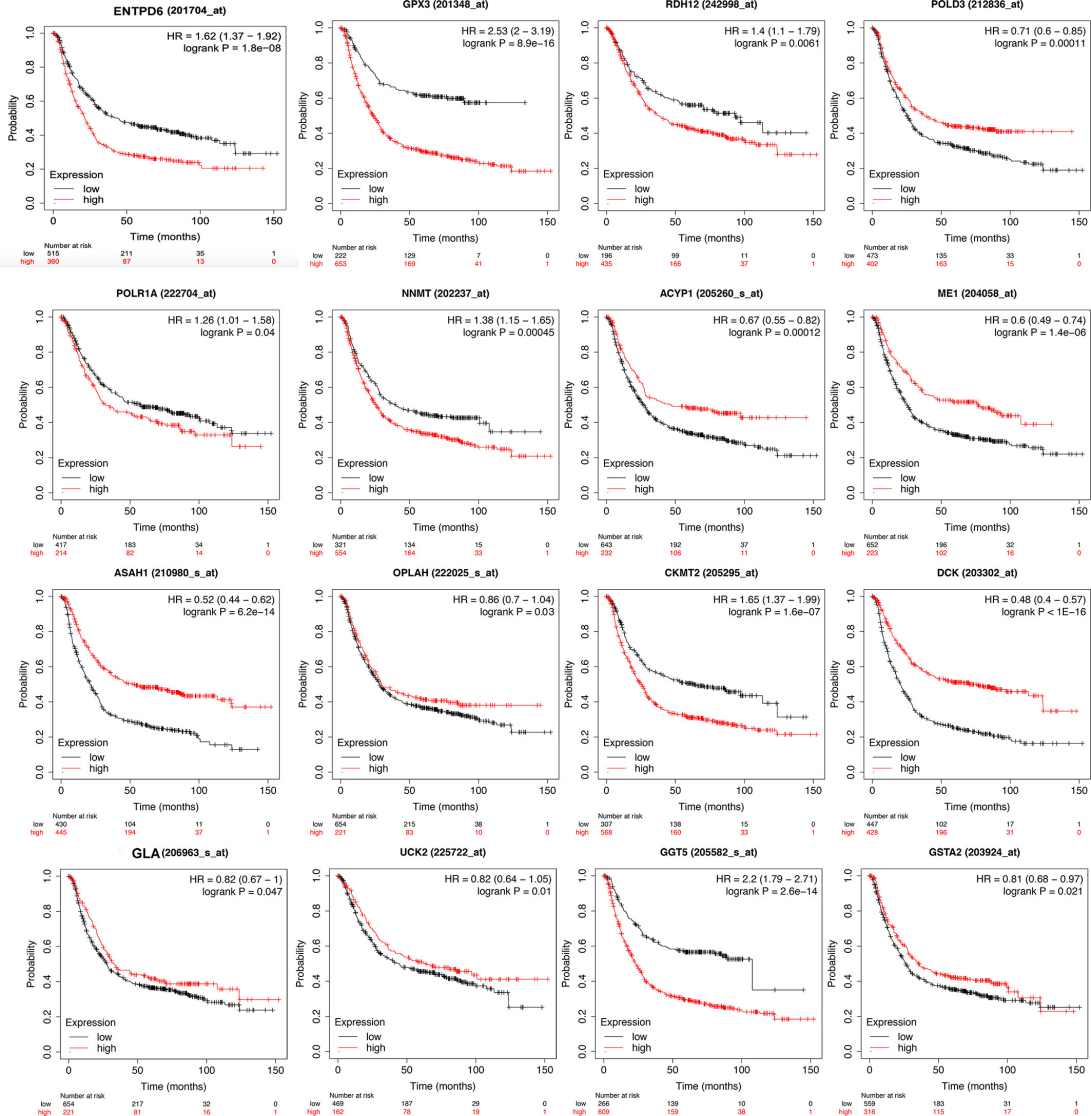
Kaplan-Meier survival curve of overall survival associated with differentially expressed metabolism-related genes (DEMRGs) in stomach adenocarcinoma (STAD). The DEMRGs associated with overall survival of patients with STAD were *ENTPD6*, *GPX3*, *GSTA2*, *POLD3*, *GLA*, *UCK2*, *GGT5*, *DCK*, *CKMT2*, *ASAH1*, *OPLAH*, *ME1*, *ACYP1*, *NNMT*, *POLR1A*, and *RDH12* (p < 0.05).

The identified DEMRGs as continuous factors were used to perform Cox regression analysis with survival information in TCGA (**Supplementary Table 7**). A total of 16 DEMRGs were significantly related to the risk ratio in STAD (**Figure 1**), including ENTPD6, GPX3, GSTA2, POLD3, GLA, UCK2, GGT5, DCK, CKMT2, ASAH1, OPLAH, ME1, ACYP1, NNMT, POLR1A, and RDH12. The survival-related DEMRGs were further plotted in Kaplan–Meier survival curve by Kaplan-Meier Plotter according to the median value of each OS-related DEMRG from Cox regression analysis (**Figure 4**).

### Construction of OS-Related DEMRG Prognostic Model for STAD

**Figure 5:**
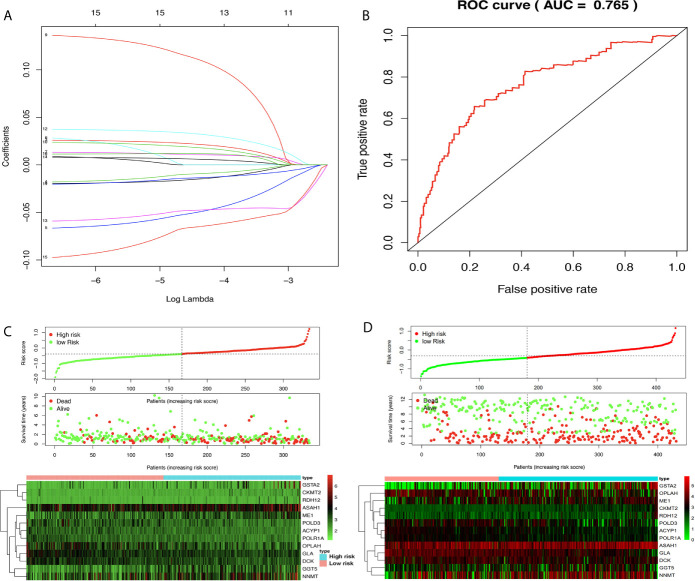
Lasso regression identified the prognostic model in adenocarcinoma (STAD). **(A)** Lasso regression complexity was controlled by lambda using the ‘glmnet’ package in R. **(B)** The receiver operating characteristic (ROC) score in STAD based on The Cancer Genome Atlas (TCGA) data. **(C)** Riskplot heatmap between high- and low-risk score groups based on TCGA data. **(D)** Riskplot between high- and low-risk score groups based on n GEO data.

The prognostic model consisting of 13 metabolism-related genes (GSTA2, POLD3, GLA, GGT5, DCK, CKMT2, ASAH1, OPLAH, ME1, ACYP1, NNMT, POLR1A, and RDH12) was constructed using lasso regression, where log (lambda) was set between −3 and −4 (**Figure 5A**). The area under the curve (AUC) value of MPRS based on the prognostic model in the TCGA group was plotted by the ROC curve, which suggested that MPRS could be a good index for evaluating the prognostic status of patients with STAD (**Figure 5B**). Additionally, the association of overall survival and MPRS was significant, which indicated that high MPRS is correlated with poor prognosis in STAD based on TCGA data (**Supplementary Table 8**). The association of overall survival and MPRS was also significant in STAD based on GEO data, which indicated that high MPRS is correlated with poor prognosis (**Supplementary Table 9**). The survival results in the TCGA and GEO groups were plotted in survival plots with gene expression (*GSTA2*, *POLD3*, *GLA*, *GGT5*, *DCK*, *CKMT2*, *ASAH1*, *OPLAH*, *ME1*, *ACYP1*, *NNMT*, *POLR1A*, and *RDH12*) heatmaps based on TCGA data (**Figure 5C**) and GEO data (**Figure 5D**).

**Figure 6:**
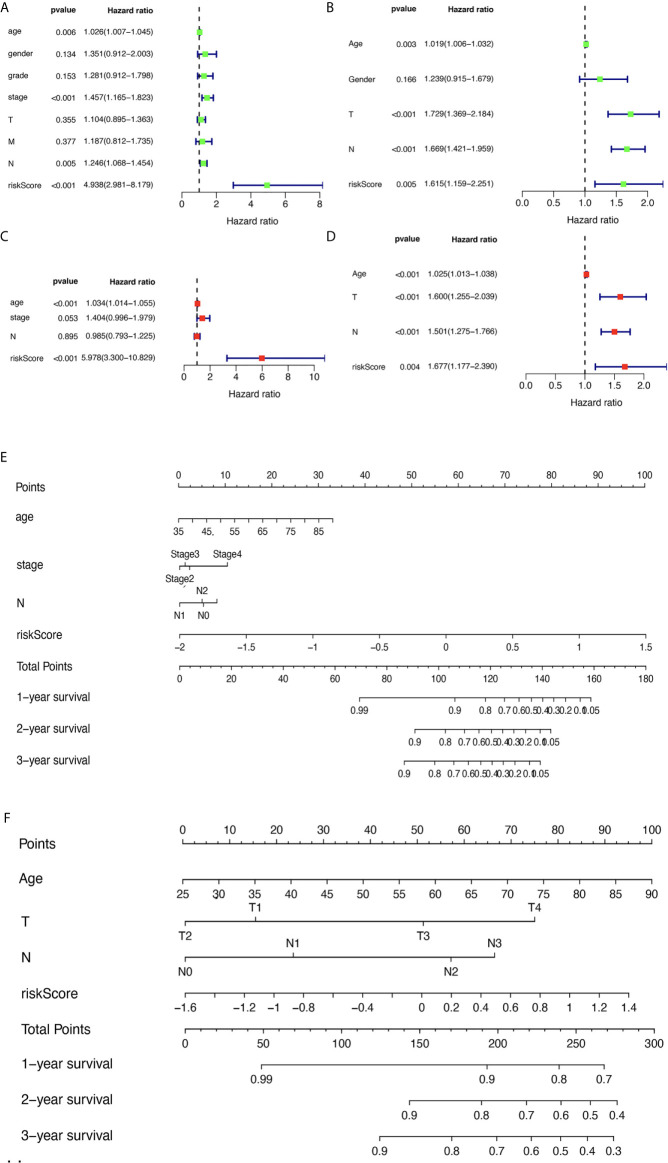
The relevance of clinical features and metabolism-related risk scores in stomach adenocarcinoma (STAD). **(A)** Univariate Cox regression analysis of risk factors in STAD based on The Cancer Genome Atlas (TCGA) data. **(B)** Multivariate Cox regression analysis of risk factors in STAD based on TCGA data. **(C)** Univariate Cox regression analysis of risk factors in STAD based on GEO data. **(D)** Multivariate Cox regression analysis of risk factors in STAD based on GEO data. **(E)** The risk score and clinical information assessment nomogram to evaluate STAD prognosis based on TCGA data (1-, 2-, and 3-year survival rates). **(F)** The risk score and clinical information assessment nomogram to evaluate STAD prognosis based on GEO data (1-, 2-, and 3-year survival rates).

The corresponding clinical data of the TCGA and GEO groups are listed in **Supplementary Table 10** and **Supplementary Table 11**. The univariate analysis revealed that age, pathologic stage, pathologic N, and risk score were significantly related to OS (**Figure 6A**) based on TCGA data. The multivariate analysis revealed that age and risk score might be independent risk factors for STAD (**Figure 6B**) based on TCGA data. To verify the results, the univariate analysis was also performed with GEO data, which revealed that age, pathologic T, pathologic N, and risk score were significantly related to OS (**Figure 6C**). The multivariate analysis was also performed with GEO data, which revealed that age, pathologic T, pathologic N, and risk score might be independent risk factors for STAD (**Figure 6D**). Furthermore, a nomogram plot was constructed to guide clinical application of basic clinical characteristics, including age at initial diagnosis, sex, pathologic M stage, pathologic T stage, pathologic N stage, pathologic stage, and MPRS to estimate patient survival (**Figure 6E** **)** based on TCGA data. The nomogram plot was also constructed using GEO data to verify the consistency of the results to estimate the patient survival rate (**Figure 6F**).

### GSEA Identified Some Significant Gene Sets Between High- and Low-MPRS Groups Based on TCGA and GEO Data

**Figure 7:**
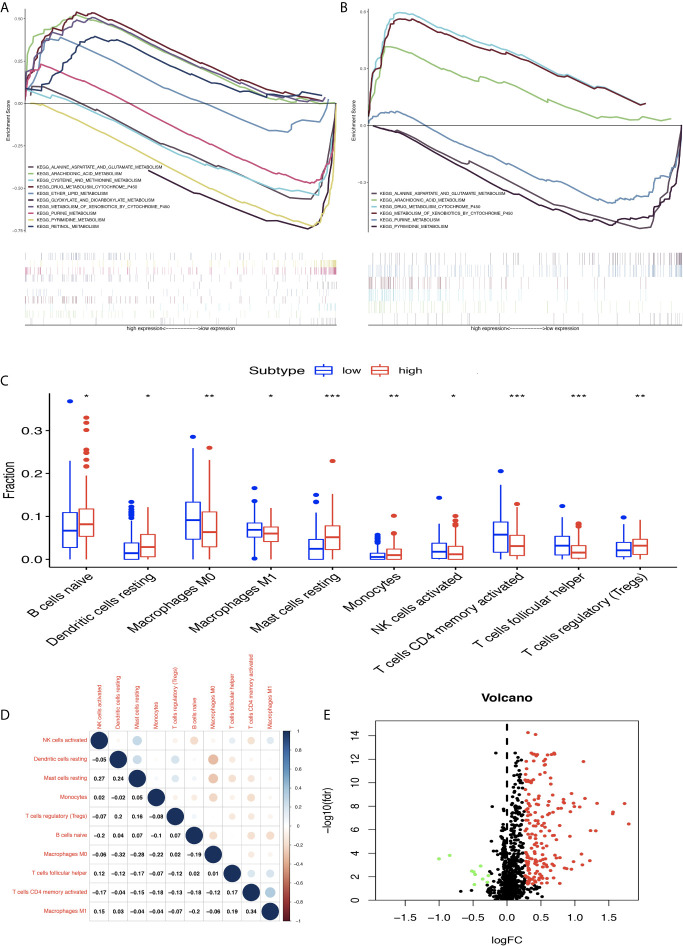
Differentially expressed immune-related genes (DEIRGs) based on Molecular Signatures Database (GSEA) data and different immune responses between high- and low-risk score groups in stomach adenocarcinoma (STAD). **(A)** DEIRGs between high- and low-risk score groups based on The Cancer Genome Atlas (TCGA) data. **(B)** DEIRGs between high- and low-risk score groups based on Gene Expression Omnibus (GEO) data. **(C)** The differential distribution of immune cells between high- and low-risk score groups. **(D)** The correlation between 10 types of immune cells in STAD. **(E)** Volcano plot of DEIRGs in STAD between high- and low-risk score groups. Upregulated DEIRGs are shown as red points, and downregulated DEIRGs are shown as green points. *p < 0.05, **p < 0.01, and ***p < 0.001.

The STAD samples were divided into two groups according to MPRS. Based on TCGA data, the significant gene sets enriched in the high-MPRS group were drug metabolism by cytochrome p450, metabolism of xenobiotics by cytochrome p450, retinol metabolism, arachidonic acid metabolism, and ether lipid metabolism and those in the low-MPRS group were cysteine and methionine metabolism, glyoxylate and dicarboxylate metabolism, purine metabolism, alanine aspartate, and glutamate metabolism, and pyrimidine metabolism (**Figure 7A** and **Supplementary Table 12**). The results were consistent with significant gene sets enriched in GEO data. Based on GEO data, the significant gene sets enriched in the high-MPRS group were drug metabolism cytochrome p450, metabolism of xenobiotics by cytochrome p450, and arachidonic acid metabolism and those in the low-MPRS group were purine metabolism, alanine aspartate and glutamate metabolism, and pyrimidine metabolism (**Figure 7B** and **Supplementary Table 12**).

### Differential Distribution of Immune Cells, Expressed IRGs, and Immune-Related Pathways Between High- and Low-MPRS Groups

**Figure 8:**
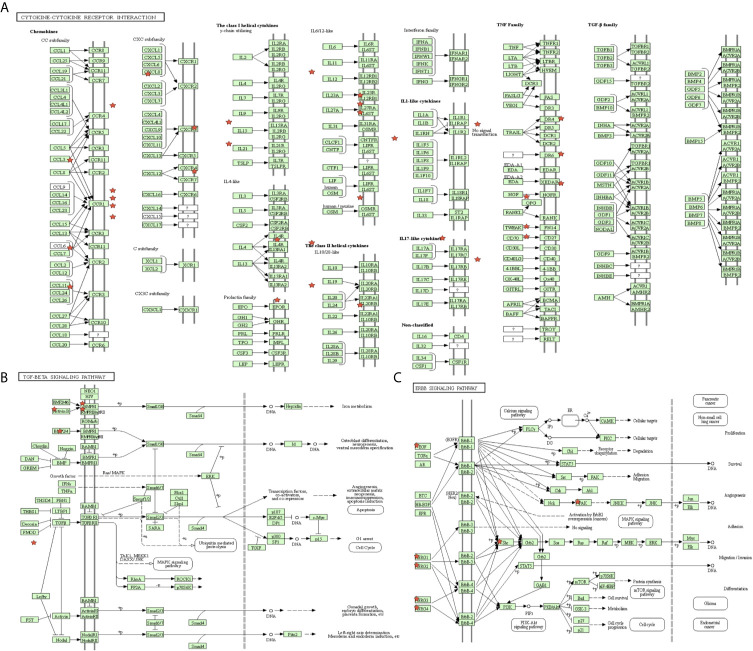
The significant immune-related pathways of differentially expressed immune-related genes (DEIRGs) in stomach adenocarcinoma (STAD). **(A)** DEIRGs significantly enriched in cytokine-cytokine receptor interaction pathway. **(B)** DEIRGs significantly enriched in TGF-β signaling pathway. **(C)** DEIRGs significantly enriched in ErbB signaling pathway.

The proportion of immune cells in STAD was significantly different between the high- and low-MPRS groups, including naïve B cells, monocytes, macrophages M0, macrophages M1, activated NK cells, Tregs, activated memory CD4 T cells, follicular helper T cells, and resting dendritic cells (**Figure 7C** and **Supplementary Table 13**). Additionally, some of the different proportions of immune cells between risk score subtypes correlated with each other; for example, M1 macrophages and activated memory CD4 T cells, M0 macrophages, and resting dendritic cells (**Figure 7D**). Furthermore, 194 DEIRGs were identified between the high-risk and low-risk score groups, including 10 downregulated and 183 upregulated IRGs (**Figure 7E** and **Supplementary Table 14**). The DEIRGs were enriched in 12 significant KEGG pathways: cytokine-cytokine receptor interaction (**Figure 8A**), TGF-beta signaling pathway (**Figure 8B**), ErbB signaling pathway (**Figure 8C**), neuroactive ligand-receptor interaction, neuroactive ligand-receptor interaction, melanoma, hematopoietic cell lineage, Jak-STAT signaling pathway, pathways in cancer, calcium signaling pathway, regulation of actin cytoskeleton, MAPK signaling pathway, and chemokine signaling pathway (**Supplementary Table 15**).

### RT-qPCR and Protein Levels Confirmed the Identified Molecules

**Figure 9:**
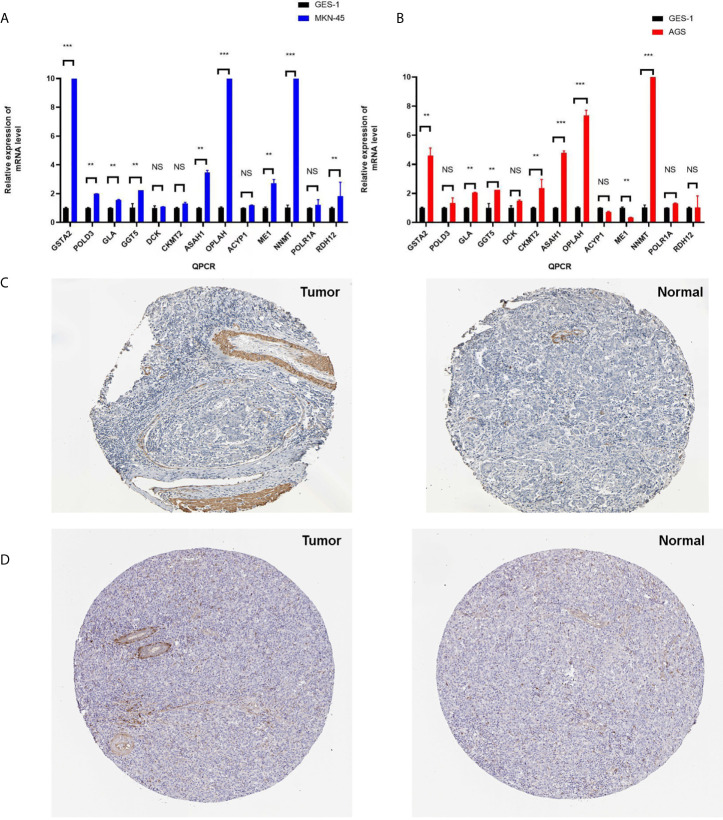
The verification of identified genes using PCR and HPA database. **(A)** Q-PCR was used to validate the expressions of 13 metabolism-related genes (GSTA2, POLD3, GLA, GGT5, DCK, CKMT2, ASAH1, OPLAH, ME1, ACYP1, NNMT, POLR1A, and RDH12) between MKN-45 and GES-1. **(B)** Q-PCR was used to validate the expressions of 13 metabolism-related genes (GSTA2, POLD3, GLA, GGT5, DCK, CKMT2, ASAH1, OPLAH, ME1, ACYP1, NNMT, POLR1A, and RDH12) between AGS and GES-1. **(C)** Protein expression level of GSTA2 was verified in the Human Protein Atlas (HPA). **(D)** Protein expression level of ASAH1 was verified in the HPA. *p < 0.05, **p < 0.01, and ***p < 0.001. NS, None significance.

Furthermore, qRT-PCR was used to validate the expressions of 13 metabolism-related genes (GSTA2, POLD3, GLA, GGT5, DCK, CKMT2, ASAH1, OPLAH, ME1, ACYP1, NNMT, POLR1A, and RDH12). The results showed that no significant difference was found for four metabolism-related genes (DCK, CKMT2, ACYP1, and POLR1A) between MKN-45 and GES-1 (**Figure 9A**). The results showed that no significant difference was found for four metabolism-related genes (GLA, DCK, ACYP1, POLR1A, and RDH12) between AGS and GES-1 (**Figure 9B**). All other genes were significantly different expressed between cancer cells and control cells. Protein expression levels were verified in the Human Protein Atlas (HPA) (https://www.proteinatlas.org/), and here two represent results were provided in **Figures 9C, D**, which indicated that the identified metabolism-related proteins were overexpressed in STAD tissues. Other protein expression levels can be checked online (https://www.proteinatlas.org/) (21).

## Discussion

Despite great improvements in the diagnosis, prevention, and treatment, patients with STAD still have a poor prognosis and an unsatisfactory survival rate (23). With the development and application of prognostic and diagnostic signatures in clinical practice, molecular biomarkers, such as methylation state non-coding RNA, and mRNA have greatly contributed to patient classification, disease status monitoring, and personalized therapeutic schedules (24). Further studies on potential molecular biomarkers will benefit patients enormously. Cancer tissues often exhibit an abnormal metabolic profile, which is known as the “cancer metabolome” (25). Some of these aberrant metabolites are significantly associated with the proliferation, progression, recurrence, and metastasis of cancer cells (26). The main metabolic hallmarks of STAD have encouraged researchers to analyze metabolites. For example, the intermediates of the glycolysis/TCA cycle have a wide range of functions in multiple cellular processes. The inhibition of the activity of the 2-oxoglutarate dehydrogenase (OGDH) complex resulted in a decrease in mitochondrial membrane potential (ΔΨm) and ATP production and an increase in ROS levels and the NADP/NADPH ratio, which affected cellular energy metabolism to suppress STAD cell growth and migration (27). The deregulated uptake of some amino acid-related metabolic enzymes also has a wide range of functions in multiple cellular processes. For example, glutaminase 1 (GLS1) and gamma-glutamylcyclotransferase (GGCT) were found to be overexpressed in patients with STAD using LC-ESI-MS/MS. The co-expression level of GLS1 and GGCT was significantly associated with lymph node metastasis, histological grade, and TNM stage in STAD (28). Other metabolic hallmarks influence cancer cells, such as increased demand for nitrogen, metabolic interactions, and alterations in metabolite-driven genes (29). With the development of immunotherapy strategies against cancer, the activity and safety of the anti-PD-1 antibody pembrolizumab have been assessed in STAD patients with PD-L1-positive recurrence or metastasis. In an open-label, multicenter, phase 1b trial, pembrolizumab showed promising anti-tumor activity and toxicity profile in patients with STAD (30). Immunometabolism is an emerging field that can provide an understanding of the association between cancer metabolism and immune response in STAD. In the tumor microenvironment, metabolic remodeling and metabolic reprogramming of immune cells promote tumorigenesis, tumor progression, treatment resistance, and metastasis (31).

In our study, we performed lasso regression analysis to construct a metabolism-related prognostic model consisting of 13 genes (*GSTA2*, *POLD3*, *GLA*, *GGT5*, *DCK*, *CKMT2*, *ASAH1*, *OPLAH*, *ME1*, *ACYP1*, *NNMT*, *POLR1A*, and *RDH12*) from 184 DEMRGs in both TCGA and GEO databases. The identification of this gene signature allowed the analysis of metabolism-related pathways and metabolic signatures at the transcriptional level to explore prognostic markers in STAD. We obtained high- and low-MPRS groups according to the metabolic prognostic signature. Furthermore, alterations in the immune cells in the tumor microenvironment between the high- and low-MPRS groups indicated an association between metabolic reprogramming and immune cells. The systematic analysis of metabolism-related genes in STAD has explored their potential roles as prognostic markers in STAD. The findings of our study were consistent with those of a previous study when we checked GenCLiP 3 (22). For example, glutaminase (GLS1), a protein associated with energy metabolism in cancer cells, encodes glutaminase, which catalyzes the hydrolysis of glutamine to glutamate and ammonia and plays a predominant role in the formation of malignant tumors. Studies on metabolic reprogramming, which targets glutamine metabolism in cancer cells, have focused on the glutaminase isozyme GLS (32). In addition, the result was also consistent with our experimental result, that glutaminase expressed higher in cancer cells (AGS and MNK-45) than normal control cells (GES-1). Lactate dehydrogenase A (LDHA) catalyzes the conversion of L-lactate and NAD to pyruvate and NADH during anaerobic glycolysis. Targeting LDHA to remodel the metabolic pathway has shown anticancer activity in cancer cells. When the function of LDHA was inhibited, energy metabolism could convert glycolysis to oxidative phosphorylation, leading to an increase in ROS levels and mitochondrial dysfunction. The potential therapeutic value of targeting metabolite-driven genes for the treatment of cancer is breaking new ground (33). *GSTM1* encodes glutathione S-transferase, and mutations in this gene have been linked to several biological processes, including drug susceptibility, oxidative stress, environmental toxicity, and tumorigenesis. A total of 237 cases and 250 controls were genotyped for the GST1 polymorphism using the PCR-RFLP technique. GST1 was identified as a prognostic marker, which is closely related to the metabolism of xenobiotics in lung cancer. Therefore, patients carrying the mutant version of *GSTM1* show the highest risk of lung cancer (34). Abnormal metabolism of choline and ethanolamine phospholipids is prevalent in almost all types of cancers. *CHKA* encodes choline kinase alpha protein, which plays a key role in the biosynthesis of phosphatidylcholine. Abnormal choline phospholipid metabolism in cancers frequently results from CHKA overexpression and hyperactivity. The novel choline kinase inhibitor could reprogram cellular metabolism and inhibit cancer cell growth (35). Among the identified 184 DEMRGs in both TCGA and GEO databases, 64 DEMRGs have been reported to be related to cancer metabolism, including *GLS*, *CYP3A4*, *HK1*, *GSTM1*, *HK*2, *LDHA*, *CHKA*, *SHMT2*, *AKR1C3*, *G6P*D, *ADH1B*, *TYMS*, *PFKP*, *RRM1*, *AKR1C2*, *SAT1*, *ODC1*, *DCK*, and *ASAH1*, *PIK3CB*, *PYCR1*, *AKR1B10*, *ACO2*, *AKR1C1*, *GPI*, *GLUL*, *NNMT*, *ALDH3A1*, *EPHX1*, *ASS1*, *CBR1*, *SMPD1*, *MTHFD2*, *TK1*, *ACSL4*, *ME1*, *AGPS*, *UGT1A10*, *MTHFD1*, *RRM2*, *EPHX2*, *LPCAT1*, *ACP5, CYP2B6, INPP5A, PAFAH1B2, ASAH2, NUDT5, MTHFD1L, PYGB, GPT, ACP1, ADH7, BLVRB, CHDH, PTGS1, GSTA2, CES1, GSTA1, DNMT1*, *ACACB, FTH1, GSS*, and *G6PC*, according to search with the keywords “metabolism and cancer” in GenCLiP 3 database (http://ci.smu.edu.cn/genclip3/input_enrichment.php#) (22). In the present study, we comprehensively examined the mRNA signature associated with STAD survival in the discovery stage (TCGA-STAD) based on RNA-Seq data and the validation stage (GEO dataset) based on microarray data. Our results showed that significant difference was found for nine metabolism-related genes (*GSTA2*, *POLD3*, *GLA*, *GGT5*, *ASAH1*, *OPLAH*, *ME1*, *NNMT*, and *RDH12*) between MKN-45 and GES-1. The results also showed that significant difference was found for eight metabolism-related genes (*GSTA2*, *GLA*, *GGT5*, *CKMT2*, *ASAH1*, *OPLAH*, *ME1*, and *NNMT*) between AGS and GES-1. The signature was first applied in the training set and was then validated in the testing set, suggesting that it was reliable. To testify the universality in different patients and to verify its application in different clinicopathological subgroups, survival analysis was performed in various subgroups. We found that the signature was independent of other potential predictors, including age, sex, stage, and grade, and its performances were of satisfaction. This suggests that most of our results were consistent with those of a previous study, with some new findings. Potential function of the mRNA encoding genes was annotated based on the gene ontology functional enrichment analysis. Among the encoding genes for 13 mRNAs significantly associated with STAD survival in the replication analysis, most of identified metabolism related genes were enriched in the metabolic process. So we think those hub genes might affect molecular metabolism, including nucleic acid, amino acid, and fatty acid, in caner related pathways. Furthermore, KEGG enrichment of DEMRGs between tumor and normal tissues in STAD showed the involvement of some significant pathways. The identified pathways influence several metabolic processes, such as ribonucleic acid metabolism (nucleotide sugar, pyrimidine, and purine), glucose and lipid metabolism (glycolysis/gluconeogenesis, fatty acid, fructose, mannose, pentose phosphate, pyruvate, and fatty acid elongation in mitochondria), amino acid metabolism (arginine, proline, alanine, aspartate, glutamate, glycine, serine, threonine, cysteine, methionine, tyrosine, tryptophan, valine, leucine, isoleucine, phenylalanine, selenoamino acid, and cyanoamino acid). These metabolism-related pathways provide clues to further studies on metabolic rewiring in cancers. For example, most cancer cells exhibit aberrant activation of lipid metabolism, which induces tumors to synthesize, elongate, and desaturate fatty acids to promote tumorigenesis, proliferation, and progression (36). Patients with upregulated glutaminolysis, glycolysis, and *de novo* synthesis of fatty acids are in a hypercatabolic state. The development of novel drugs targeting cancer anabolism or host catabolism has made great achievements in anticancer experimental treatments (37). Additionally, hub molecules were obtained from the PPI network, and their functions were evaluated in further studies. Some of these genes have been reported to be crucial in cancer metabolism. For example, glucose-6-phosphate dehydrogenase (G6PD) produces key electron donors, such as NADPH, against oxidizing agents. The oxidative pentose-phosphate pathway maintains a normal NADPH/NADP ratio to support cell growth. All the molecules involved in the oxidative pentose phosphate pathway are important for cell growth. Loss of G6PD in cancer cells generates high NADP, induces compensatory increases in malic enzyme 1 and isocitrate dehydrogenase, and inhibits dihydrofolate reductase activity to block folate-mediated biosynthesis (37). Cytochrome P450 (CYP3A) proteins are involved in the metabolism of approximately half the drugs, such as cyclosporin A, acetaminophen, diazepam, codeine, and erythromycin. Cytochrome P450 also metabolizes carcinogens, steroids, and other lipids. The polymorphisms CYP3A5*3 and CYP3A4* 1 B were tested more frequently in patients with primary lung tumors than in normal volunteers. The CYP3A5*3/4* 1B genotype might have high levels of CYP3A4 activity, which is crucial for the biotransformation of numerous anticancer agents and the metabolism of carcinogens (38). In our study, the identification of meaningful OS DEMRGs in STAD and their enriched pathways involved in the development and progression of SATD would provide valuable prospects for clinical diagnosis and new therapeutic strategies. However, further study and verification of the identified OS-DEMRGs in a prognostic model are necessary.

Along with the advancement in tumor immunology, immunotherapy combined with other therapies against tumors has been applied in clinical practice (39). Increasing evidence suggests that metabolic remodeling and metabolic reprogramming play a crucial role in the immune response to affect tumorigenesis, progression, invasion, and metastasis in various cancer cells (40). However, studies on the association between metabolic processes and the immune system (immune-related genes and pathways) are limited, which has hindered the advancement in the clinical application of combined metabolism-targeting drugs and immune checkpoint inhibitors (41). Previous studies have shown that immune cell responses and metabolism signaling networks are dynamically regulated. For example, serine/threonine kinase-mediated signaling networks can act as upstream regulators to regulate the metabolism of T cells. Immunometabolic signaling networks may uncover more therapeutic possibilities targeting metabolic molecules and immune cell responses in human cancers (42). In this study, the proportion of immune cells in STAD was significantly different between the high- and low-MPRS groups, including naïve B cells, monocytes, M0 macrophages, M1 macrophages, activated NK cells, Tregs, activated memory CD4 T cells, follicular helper T cells, and resting dendritic cells. Furthermore, the 194 DEIRGs were enriched in 12 significant KEGG pathways. In this study, we investigated some of the immune-related genes driven by metabolism in depth. For example, evidence has shown that HIF-1α/LDH-A mediates cell metabolism by causing a shift between aerobic glycolysis and oxidative phosphorylation, which alters PD-L1 expression; thus, the upregulated expression of checkpoint inhibitor PD-L1 induces tumor resistance to therapy (43). *GHRL* was one of the DEIRGs identified in our study, which acts as a powerful appetite stimulant and plays a key role in energy homeostasis. GHRL can regulate whole-body metabolism *via* the ghrelin-signaling pathway in the hypothalamus and alter the metabolic activity of cancer and immune cells (44). A systematic analysis of immune-related genes between high- and low-MPRS subtypes to clarify the role of metabolism in cancer immunotherapy would be meaningful.

## Conclusion

The findings of our study were consistent with the previous study, but we focused on the cross-talking between metabolic reprogramming and immune system (45). In summary, we performed a systematic analysis of metabolism-related genes for predicting the prognosis of STAD, constructed a 13-gene metabolic signature as a prognostic model, and explored the association between metabolism and cancer immunity. The identified OS-related DEMRGs, DEIRGs, enriched metabolism-related pathways, and enriched immune-related pathways may play an important role in STAD tumorigenesis and deserve further study in clinical applications as diagnostic biomarkers and therapeutic targets.

## Data Availability Statement

The datasets presented in this study can be found in online repositories. The names of the repository/repositories and accession number(s) can be found in the article/**Supplementary Material**.

## Author Contributions

ZY designed the study, analyzed the data, prepared figures and tables, and wrote the manuscript. LC conceived the study, supervised the results, critically revised and wrote the manuscript, and was responsible for its financial support and the corresponding works. MZ, YZ, SW and HH analyzed the data and conceived the study. QL, YW, ZL, SC and QZ critically revised the manuscript. ZY, MZ, YZ, SW and HH contributed equally to the work. All authors contributed to the article and approved the submitted version.

## Funding

This research was sponsored by the National Clinical Key Specialty Construction Program of China and grants from the National Science Foundation Project of Fujian Science and Technology Department (no. 2017J01264 and no. 2018Y0015), Foundation for Fujian Provincial Health Technology Project (nos. 2019-ZQN-16, 2019-CXB-9, 2019006), and Foundation of Fujian Maternity and Child Health Hospital (No.YCXM 18-09).

## Conflict of Interest

The authors declare that the research was conducted in the absence of any commercial or financial relationships that could be construed as a potential conflict of interest.
